# Delayed Cervical and Multifocal Spinal Metastases From Rectal Adenocarcinoma: A Case Report of Severe Canal Stenosis, Neurological Decline, and Septic Shock

**DOI:** 10.7759/cureus.115935

**Published:** 2026-09-08

**Authors:** Sandra Porter-Edwards, Lavon Edwards

**Affiliations:** 1 Health Education, Kaiser Permanente, Atlanta, USA; 2 Medicine, Columbus Central University School of Medicine, Atlanta, USA; 3 Clinical Science, TrueMed Health Institute, Atlanta, USA

**Keywords:** anemia, malnourished, neurological decline, post colostomy, rectal adenocarcinoma, spinal canal stenosis, spinal metastases

## Abstract

A 76-year-old male with a past medical history significant for rectal adenocarcinoma diagnosed in 2014 presented to the emergency department with worsening left arm pain, generalized weakness, and two syncopal episodes. The patient previously underwent sigmoid colectomy followed by low anterior resection and permanent colostomy. Imaging revealed diffuse spinal metastases with extensive epidural tumor causing multilevel severe canal stenosis and spinal cord compression. Additional findings included hepatic metastases, bilateral pleural effusions, supraclavicular nodal disease, and suspected leptomeningeal involvement. This case highlights the rare but devastating pattern of colorectal cancer metastasis to the spine and underscores the importance of early recognition of metastatic epidural disease, multidisciplinary management coordination, palliative care integration.

## Introduction

Despite significant advances in screening, surgical management, and systemic therapies, colorectal cancer (CRC) remains one of the leading causes of cancer-related morbidity and mortality worldwide [1]. The liver and lungs represent the most common sites of distant metastasis; however, osseous metastases occur far less frequently and are typically associated with advanced-stage disease and poor prognosis [2,3]. Among skeletal sites, the vertebral column is the most commonly involved location due to its extensive venous communication with the systemic circulation through Batson’s vertebral venous plexus [4]. Due to the nature of clinical signs and symptoms of bone destruction, infectious processes, such as spinal epidural abscess, systemic sepsis, vertebral osteomyelitis, and discitis, may present with symptoms that closely resemble metastatic spinal disease [5]. Early differentials are essential to avoid neurological decline and increased morbidity. 

Spinal metastases from CRC are clinically significant because epidural tumor extension may result in malignant spinal cord compression (MSCC), a neurologic emergency associated with irreversible neurologic deficits, paralysis, and substantial decline in quality of life if not rapidly identified and treated [5,6].

Standard management of metastatic spinal cord compression includes urgent magnetic resonance imaging (MRI), corticosteroid therapy when appropriate, surgical decompression in selected patients, radiation therapy, and systemic treatment based on tumor biology and the patient’s functional status [7,8]. Bone metastases in colorectal cancer, compared with hepatic and pulmonary metastases, are not very common; however, when present, they most commonly involve the vertebral column and present with substantial pain, neurologic issues, and reduced survival rates; in turn, prompt intervention is needed for preventative measures and early detection [9]. Patients presenting with vertebral involvement require rapid evaluation for spinal abnormalities or metastatic spinal cord compression. This is followed by individualized treatment, which typically involves corticosteroids, radiotherapy, surgical decompression, systemic therapy, and early palliative care integration [10]. Currently, there are various treatment options available, and new developments are emerging in whole-exome sequencing [11]. Routine surveillance following treatment for rectal adenocarcinoma, including clinical evaluation, carcinoembryonic antigen (CEA) monitoring, colonoscopy, and cross-sectional imaging, is essential for early detection of recurrent disease and development of symptomatic metastatic progression [8,11].

Cervical and multifocal spinal involvement are particularly uncommon manifestations and often occur late in the disease course, sometimes years after presumed remission or curative treatment [11]. Patients with rectal adenocarcinoma must follow established surveillance protocols in order to detect recurrence prior to the onset of symptomatic metastatic disease. Regular clinical assessments, surveillance colonoscopies, periodic cross-sectional imaging, and CEA monitoring are usually advised forms of follow-up.

Early detection of recurrence may allow for the start of systemic therapy or potentially curative interventions before widespread metastatic disease develops. Molecular profiling is increasingly used in the management of metastatic colorectal cancer to inform treatment choices. RAS, BRAF, HER2, microsatellite instability (MSI), mismatch repair (MMR), and Neurotrophic Tyrosine Receptor Kinase (NTRK) status are examples of biomarkers that can affect therapeutic decision-making and targeted therapy eligibility. Modern systemic treatment approaches for patients with metastatic disease may include anti-vascular endothelial growth factor (VEGF) treatments like bevacizumab, anti-epidermal growth factor receptor (EGFR) treatments for RAS wild-type tumors, immunotherapy for MSI-high or deficient mismatch repair tumors, and fluoropyrimidine-based chemotherapy in combination with oxaliplatin or irinotecan. Recurrent disease can be better controlled, functional status can be maintained, and survival can be increased with early detection and prompt systemic therapy initiation [11]. 

Infectious pathogens may further complicate the management of advanced colorectal cancer. Disruption of the intestinal mucosal barrier by advanced malignancy or bowel perforation can facilitate translocation of enteric organisms, such as *Escherichia coli*, into the bloodstream. As a Gram-negative bacterium, *E. coli* produces lipopolysaccharide (LPS) endotoxin, which can trigger a systemic inflammatory response resulting in septic shock, multiorgan dysfunction, and high mortality, particularly in patients with advanced gastrointestinal malignancies [12].

We report a rare case of delayed metastatic rectal adenocarcinoma presenting nearly a decade after initial diagnosis with diffuse spinal epidural disease involving the cervical, thoracic, and lumbar spine. This resulted in severe canal stenosis, cord compression, and suspected leptomeningeal dissemination. In the case of malignancy, TNF-*α* and IL-1 cause hypotension, increased vasodilation, and vascular permeability. The patient’s clinical course was further complicated by septic shock due to *E. coli*, which ultimately proved fatal. This case highlights the diagnostic and therapeutic challenges of managing advanced metastatic spinal disease in the setting of life-threatening infection.

## Case presentation

A 76-year-old male with a past medical history significant for rectal adenocarcinoma diagnosed in 2014 presented to the emergency department with worsening left arm pain, generalized weakness, and two syncopal episodes. The patient previously underwent sigmoid colectomy followed by low anterior resection and permanent colostomy. He also had a history of osteoarthritis, normocytic normochromic anemia of chronic disease, and severe cachexia with a body mass index (BMI) less than 18. 

On arrival, vital signs revealed a temperature of 38.9 °C, blood pressure 92/60 mmHg, pulse 116 bpm, and oxygen saturation 90% on room air. Physical examination demonstrated 2/5 strength in the lower extremities, diminished sensation below the T10 level, and brisk reflexes with clonus. The patient was somnolent but arousable. Laboratory findings showed leukocytosis (WBC = 17.4 × 10⁹/L), elevated lactate (4.2 mmol/L), and rising creatinine (2.1 mg/dL). Blood cultures grew *E. coli* within 12 hours (Table 1). 

**Table 1:** Vital signs and laboratory findings on presentation

Parameter	Patient Value	Reference Range	Units	Interpretation
Temperature	38.9	36.5–37.5	°C	Elevated
Blood Pressure	92/60	90/60–120/80	mmHg	Hypotensive
Heart Rate (Pulse)	116	60–100	bpm	Tachycardic
Oxygen Saturation	90	≥95	%	Reduced
White Blood Cell Count (WBC)	17.4	4.0–11.0	×10⁹/L	Leukocytosis
Lactate	4.2	0.5–2.0	mmol/L	Elevated
Creatinine	2.1	0.6–1.3	mg/dL	Elevated
Blood Cultures	Positive for *Escherichia coli* within 12 hours	Negative	—	Consistent with bacteremia/sepsis

On examination, the patient appeared febrile. Neurologic evaluation demonstrated generalized weakness with limited mobility of the left upper extremity due to pain. Respiratory examination revealed diminished breath sounds bilaterally. Initial laboratory findings were notable for anemia. His current medications included gabapentin 100 mg three times daily, oxycodone 5 mg every six hours as needed for severe pain, and sodium chloride 1 g twice daily. MRI of the cervical spine demonstrated extensive epidural tumor involvement extending from C3-C5 with severe canal stenosis at C4-C5, associated cord distortion, and subtle T2 hyperintensity concerning for cord edema. Tumor extension into the left neural foramina was present from C3-C6. MRI of the thoracic spine demonstrated epidural metastatic disease at T4 and circumferential epidural disease at T9-T10 resulting in severe canal stenosis, cord flattening, and edema extending to the conus. MRI of the lumbar spine demonstrated ventral epidural tumor extending from L1-L5 with severe multilevel canal stenosis and findings concerning for leptomeningeal disease at L3-L4. Additional imaging demonstrated bilateral pulmonary opacities, pleural effusions, and an elevated hemidiaphragm in the setting of advanced metastatic disease (Figure 1).

**Figure 1:**
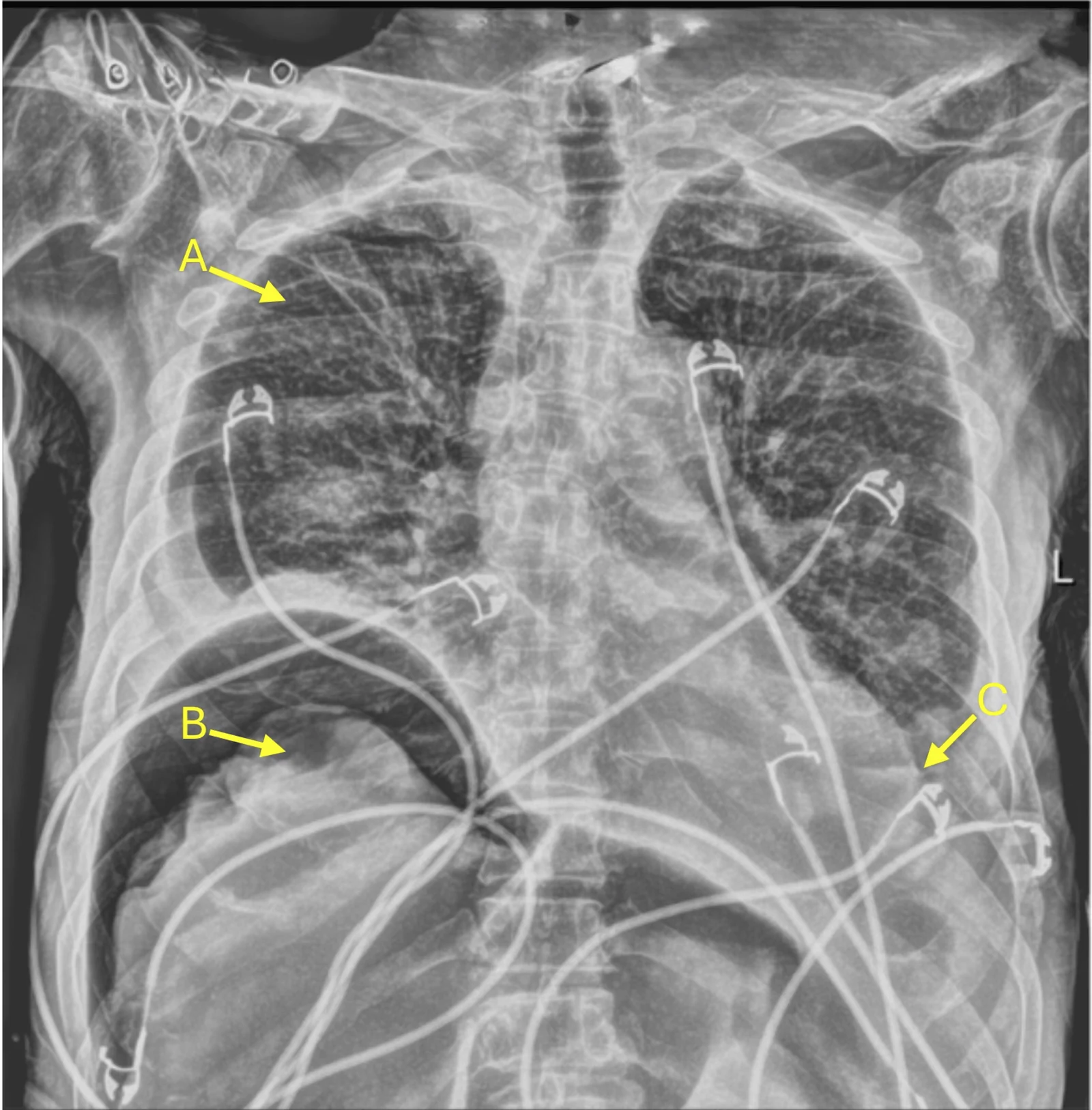
Portable chest radiograph demonstrating bilateral pulmonary opacities, pleural effusions, and elevated hemidiaphragm in the setting of metastatic disease A. Pulmonary metastases (opacities); B. Pleural effusions; C. Elevated hemidiaphragm

The imaging findings in this patient were characteristic of advanced metastatic spinal disease, demonstrating diffuse osseous metastases with multifocal epidural tumor extension involving the cervical, thoracic, and lumbar spine, severe spinal canal stenosis, neural foraminal involvement, and probable spinal cord compression. MRI plays an important role and serves as the gold standard in the evaluation of spinal metastatic disease because of its ability to analyze bone marrow involvement and assess epidural and soft tissue extension, spinal cord compression, and other areas of neural involvement. MRI findings can also assist in treatment planning [13]. The extensive cervical involvement is not a very common area of involvement. Given the uncommon metastatic sites, whole-spine MRI is important for identifying problematic lesions. Axial CT imaging of the upper cervical spine further demonstrated destructive osseous metastatic changes involving the cervical vertebrae (Figure 2). Following the description of the cervical CT findings, please add: “Contrast-enhanced CT of the neck additionally demonstrated metastatic soft tissue extension (Figure 3).

**Figure 2:**
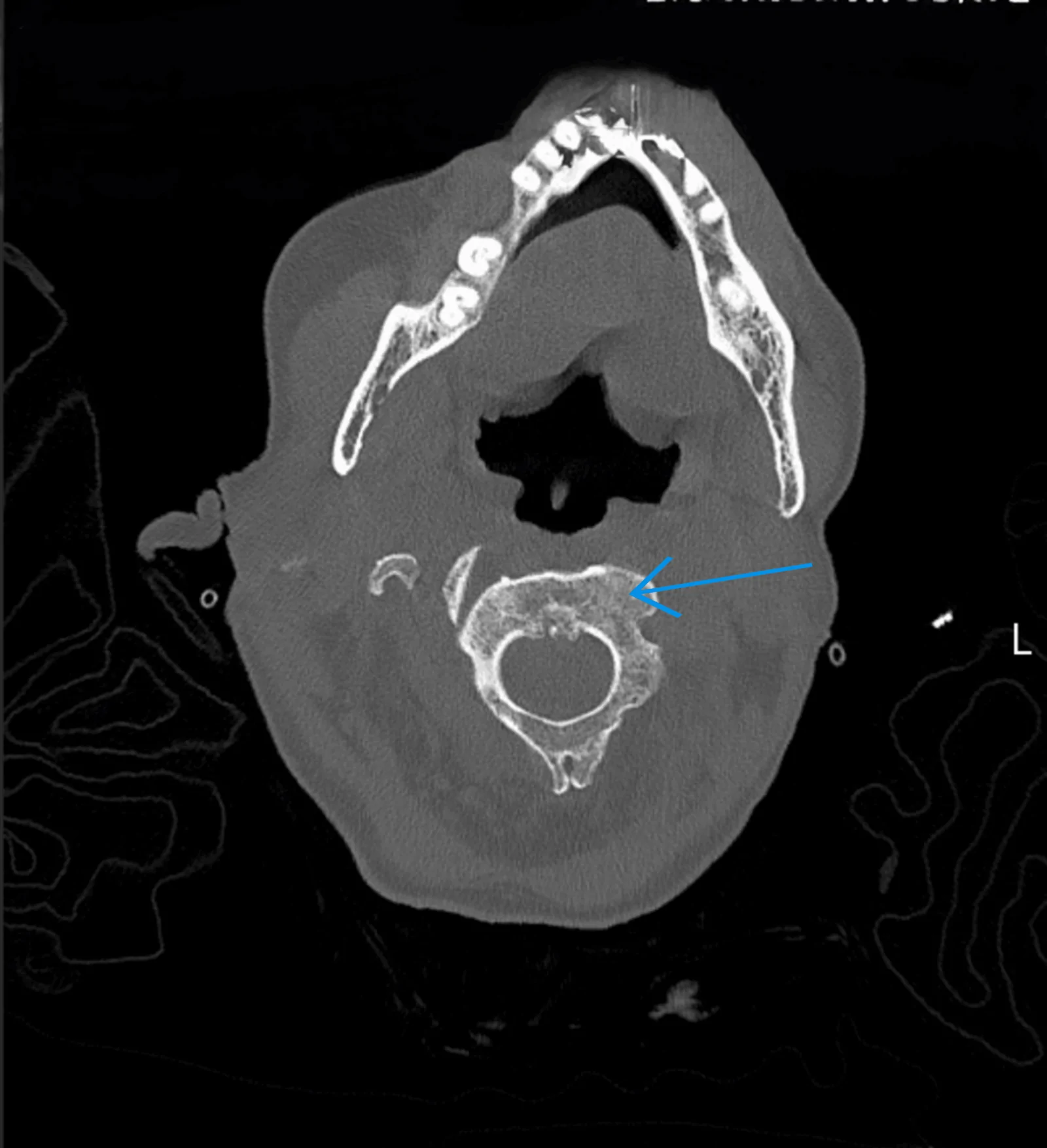
Axial CT image of the upper cervical spine demonstrating destructive osseous metastatic changes involving the cervical vertebrae

**Figure 3:**
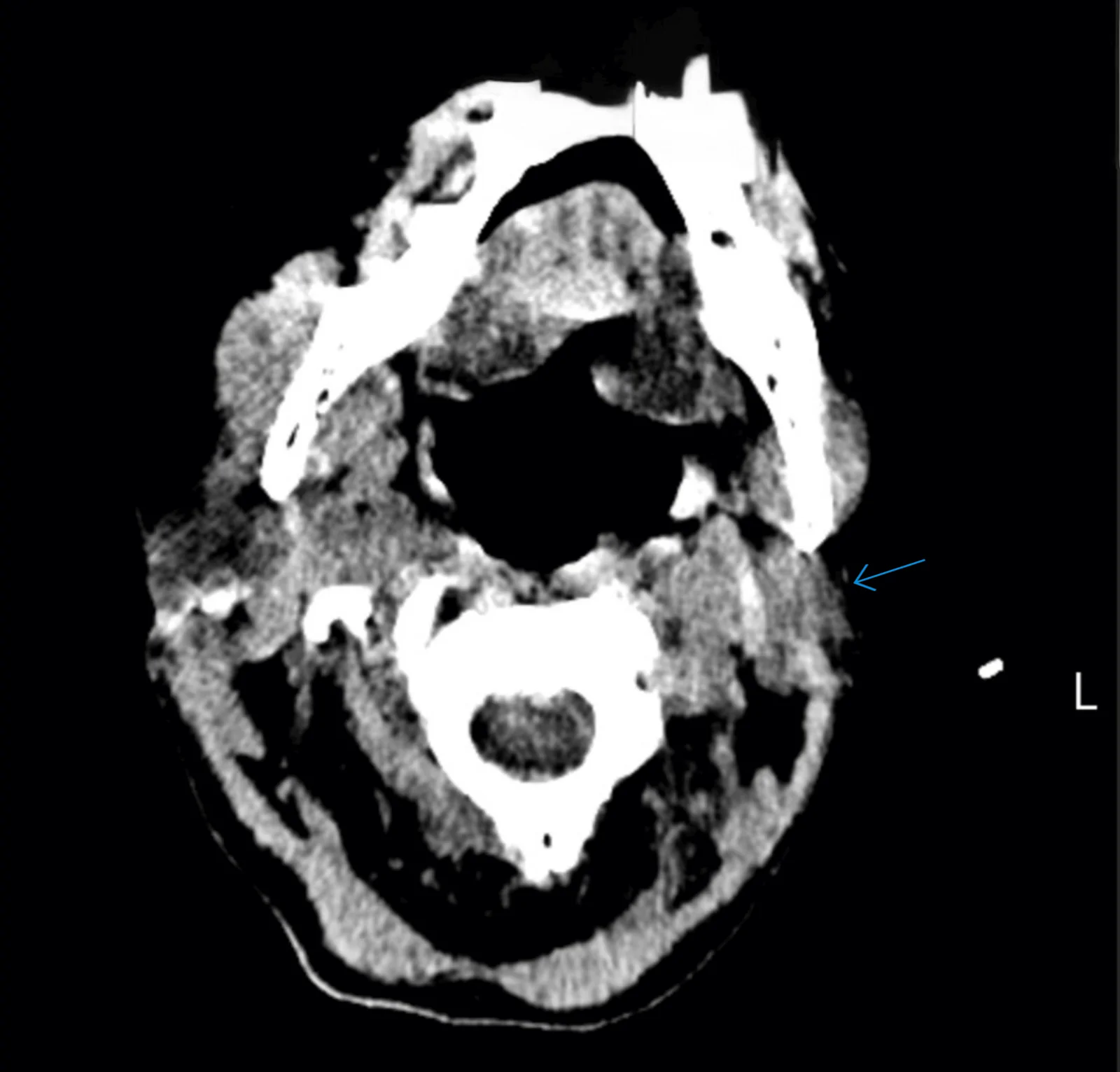
Contrast-enhanced CT neck image demonstrating metastatic soft tissue extension

Additional findings included bilateral pleural effusions, intrahepatic metastases, supraclavicular nodal metastatic disease, and sigmoid colon wall thickening. The patient was evaluated by oncology, radiation oncology, neurosurgery, and palliative care. Given the extent of metastasis and poor functional status, management focused on palliative radiation therapy integration, corticosteroids for spinal cord edema, and pain control. Despite supportive measures, including intravenous fluid resuscitation, electrolyte correction with normal saline administration, broad-spectrum antimicrobial therapy, and intensive monitoring, the patient experienced progressive clinical deterioration and subsequently died from complications of *E. coli* septic shock. 

## Discussion

Bone and spinal metastases from colorectal cancer are uncommon and generally indicate advanced systemic disease with poor prognosis [2,3]. One proposed mechanism of spread involves the vertebral venous (Batson’s) plexus, which permits hematogenous dissemination to the spine while bypassing hepatic filtration [4]. Spinal involvement is clinically significant because epidural tumor extension may lead to malignant spinal cord compression, a neurologic emergency associated with irreversible neurologic deficits if untreated [5]. Healthcare providers and clinicians should consider infectious etiologies when patients present with back pain, neurological deficits, spinal lesions, and swelling. Early diagnosis and prompt intervention are therefore critical to preserving neurologic function and improving patient outcomes.

This case highlights a rare and clinically challenging presentation of metastatic colorectal adenocarcinoma characterized by extensive multifocal spinal metastases with significant cervical spine involvement, progressive neurologic deterioration, and concurrent septic shock secondary to *E. coli* bacteremia. While osseous metastases from colorectal cancer are uncommon compared with hepatic and pulmonary metastases, cervical spine involvement remains particularly rare and carries a substantial risk of spinal instability, spinal cord compression, and irreversible neurologic deficits.

Several features distinguish this case from previously reported presentations. First, the patient demonstrated widespread metastatic involvement of the vertebral column, including the cervical spine, resulting in progressive neurologic compromise and concern for impending spinal cord compression. Second, the patient’s clinical course was further complicated by severe *E. coli* sepsis that progressed to septic shock, creating competing therapeutic priorities between infection management, oncologic treatment, and neurologic preservation. Finally, the coexistence of advanced metastatic disease, suspected bowel perforation, and profound physiological decline significantly limited available therapeutic interventions.

*E. coli* is a Gram-negative bacillus and one of the most common causes of bloodstream infections in patients with gastrointestinal malignancies. Disruption of the intestinal mucosal barrier by advanced colorectal cancer, bowel perforation, immunosuppression, or invasive procedures may facilitate bacterial transfer into the bloodstream. Once systemic dissemination occurs, endotoxin-mediated inflammatory responses can precipitate septic shock, characterized by profound hypotension, tissue hypoperfusion, multiorgan dysfunction, and high mortality despite aggressive treatment. Our patient developed these complications before ultimately succumbing to terminal septic shock.

From a clinical perspective, this case emphasizes the importance of vigilant surveillance in patients with a history of colorectal cancer, particularly when new neck pain, back pain, weakness, sensory deficits, or gait disturbances develop. Early recognition of spinal metastatic disease may facilitate timely intervention before irreversible neurologic injury occurs. Sepsis can occur concurrently with advanced malignancy or drive spinal infections. This overlap highlights the necessity of a thorough diagnostic workup, including urgent laboratory evaluation, blood cultures, inflammatory markers, and advanced imaging. Furthermore, comprehensive management of metastatic colorectal cancer increasingly relies on molecular profiling and individualized systemic therapies, including chemotherapy, anti-VEGF agents, EGFR-targeted therapies, immunotherapy in selected molecular subtypes, and other targeted treatments [6]. Early identification of recurrent disease through routine surveillance imaging, CEA monitoring, and adherence to follow-up recommendations may improve opportunities for treatment and symptom control.

In this case, the patient demonstrated multilevel epidural metastatic disease with spinal cord compression involving the cervical, thoracic, and lumbar spine. Additional findings included hepatic metastases, pleural effusions, severe cachexia, and suspected leptomeningeal spread, all of which reflected extensive late-stage disease progression and significantly worsened prognosis. Early integration of palliative care is essential to optimize symptom management, quality of life, and goals-of-care discussions.

Clinical management and palliative care

Management of malignant spinal cord compression requires prompt intervention to prevent irreversible neurologic deterioration. Corticosteroids, particularly high-dose dexamethasone, are typically initiated rapidly to reduce spinal cord edema and inflammation, thereby alleviating neurologic compromise [7]. Palliative radiotherapy is an important treatment option for patients with multifocal disease or those who are not candidates for surgical intervention [8]. In addition, systemic therapies, including chemotherapy, targeted therapy, immunotherapy, and hormone therapy, may be used to control tumor progression. At the same time, ablative techniques and minimally invasive procedures can provide additional options for tumor control and symptom management. Collectively, these approaches aim to reduce tumor burden, alleviate pain, preserve neurologic function, and improve survival and quality of life [9]. In this patient, the extent of metastatic burden and poor functional status precluded aggressive surgical intervention. Management therefore focused primarily on corticosteroid therapy, pain control, supportive care, and family counseling.

Prognosis and lessons

Management of this patient required a multidisciplinary approach involving oncology, neurosurgery, infectious disease, critical care, and palliative care specialists. Standard management of metastatic epidural spinal cord compression includes urgent magnetic resonance imaging, corticosteroid administration when appropriate, neurosurgical evaluation, and consideration of palliative radiation therapy to preserve neurologic function and alleviate pain. However, in this case, the patient’s rapidly deteriorating clinical status and septic shock substantially limited the feasibility of aggressive oncologic or surgical interventions. Surgical management of the suspected colonic perforation was also deemed inappropriate because of the patient’s poor functional status, extensive metastatic burden, and prohibitive operative risk.

Once neurologic deficits become established, particularly in the setting of concurrent leptomeningeal and visceral metastases, meaningful neurologic recovery is unlikely. Median survival following diagnosis of spinal metastases from colorectal cancer has been reported to range from approximately 3-12 months depending on disease burden and performance status [10]. This case highlights the importance of maintaining clinical vigilance for new neurologic or musculoskeletal symptoms in colorectal cancer survivors, even many years after initial treatment and presumed remission.

Limitations

This case report has several limitations. At the time of the manuscript preparation, the patient’s original imaging archive, including high-resolution DICOM images, was not available. Hence, the lack of a complete imaging dataset limited the ability to annotate radiographic findings. Despite these limitations, the available clinical documentation, imaging interpretations, and hospital records provided sufficient evidence to characterize the patient’s disease course, diagnostic findings, management, and outcome.

## Conclusions

In the present case, MRI demonstrated extensive epidural metastatic disease extending across multiple spinal levels with severe canal stenosis and findings concerning for cord compression and cauda equina syndrome. These radiographic findings correlated closely with the patient’s progressive neurological decline and functional deterioration. Although surgical decompression is often considered for selected patients with metastatic spinal cord compression, the patient’s advanced metastatic burden, poor functional status, and subsequent septic shock precluded definitive surgical intervention.

This case demonstrates that when dealing with cases of rectal adenocarcinoma or other forms of cancer, the possibility of metastasis cannot be ignored, irrespective of the interval between the initial treatment and the onset of symptoms or pain. This case further illustrates the importance of early goals-of-care discussions in patients with advanced metastatic cancer. When prognosis becomes limited, and treatment options are constrained by severe comorbidities such as septic shock, patient-centered decision-making and integration of palliative care services become essential to ensure that treatment strategies align with patient values, symptom management priorities, and quality-of-life considerations. Without appropriate management, spinal metastases may progress and lead to infections and metastatic adenocarcinoma. Timely and appropriate management is critical to preserving neurological function, improving quality of life, and optimizing clinical outcomes.
